# The influence of control beliefs on the cardiovascular fitness of college students: the chain mediating effect of subjective exercise experience and exercise adherence

**DOI:** 10.1186/s12889-023-17509-3

**Published:** 2024-01-02

**Authors:** Hewu Lv, Ting Zhang, Bo Li, Rui Wang

**Affiliations:** 1 College of Sports Science, Nantong University, Nantong, 226019 China; 2 School of Physical Education, Central China Normal University, Wuhan, 430079 China; 3 Student Affairs Office, Nantong University, Nantong, 226019 China

**Keywords:** Physical activity, College students, Cardiovascular fitness, Control beliefs, Subjective exercise experience, Exercise adherence

## Abstract

**Purpose:**

Lack of adequate physical exercise is the main reason for the frequent occurrence of health problems among Chinese college students. The purpose of this study is to explore the effects of control beliefs on cardiovascular fitness among college students and the mediating role of subjective exercise experience and exercise adherence in it.

**Methods:**

The Control Belief Scale, the Subjective Exercise Experience Scale (SEES), and the Exercise Adherence Scale were used to investigate 1854 freshmen and sophomores in Nantong and Suzhou, China. Cardiovascular fitness data for college students from the National Student Physical Health Standard and SPSS 23.0 statistical analysis software were used to carry out statistics and analyses on the questionnaires. Correlation analysis, regression analysis, and mediation models were used to assess control beliefs, subjective exercise experiences, exercise adherence, and cardiovascular fitness.

**Results:**

The control belief of college students was directly related to cardiovascular fitness (effect value: 0.121), the mediating effect through subjective exercise experience was not significant, indirectly related through the mediating effect of exercise adherence (effect value: 0.101), and indirectly related through the mediating effect of subjective exercise experience and exercise adherence (effect value: 0. 019). The positive prediction effect of control belief on cardiovascular fitness of college students was significant (β = 0.267, *P* < 0.001), and the positive prediction effect of control belief on cardiovascular fitness of college students was still significant (β = 0.121, *P* < 0.01) after adding the intermediary variables (subjective exercise experience and exercise adherence).

**Conclusions:**

The cardiovascular fitness of college students was not only directly affected by control beliefs but also affected by the chain mediating effect of subjective exercise experience and exercise adherence. Therefore, it is necessary to improve the control beliefs, subjective exercise experiences, and exercise adherence of college students to improve their cardiovascular fitness level and enhance their physical health.

## Background

Worldwide, cardiovascular fitness has always been a hot topic, cardiovascular fitness is an important element of one’s life health [1]. Cardiovascular health reflects the capacity of the cardiovascular system, which has an important relationship with many chronic diseases such as hypertension, coronary heart disease and atherosclerosis, and is an important dimension of physical health [2]. If college students can maintain a high level of cardiovascular fitness they will not feel overtired in heavy and tense daily activities, there will be enough vitality to pursue leisure and enjoyment [3]. On the contrary, the decline of cardiovascular fitness will lead to a series of problems, such as cardiovascular disease [4], obesity [5], type 2 diabetes [6, 7], hypertension [8], sleep apnea syndrome [8], mental health [9] and so on. A decline in the cardiovascular fitness indicator was observed between 1985 and 2014 [10], the declining cardiovascular fitness of contemporary college students has caused widespread concern in society [11]. This scenario may be affected by the control belief, subjective exercise experience, and exercise adherence of college students.

Control beliefs can play a crucial role in motivating college students to engage in regular physical activity and promoting cardiovascular fitness. Control beliefs refer to an individual’s perception of their ability to control their behavior and achieve desired outcomes (e.g., self-control, self-efficacy and self-concept) [12]. According to control value theory, control beliefs are an individual’s subjective evaluation of his or her behavior and his or her ability to influence outcomes, mainly in the form of outcome expectations, self-efficacy, success and failure attributions, and self-concept [13]. Body self-concept and self-efficacy are important components of control beliefs, reflecting a person’s control beliefs regarding body perception [14], and have been found to be motivating factors for college students to consistently engage in exercise [15] and are positively associated with cardiovascular fitness in college students [16]. In light of that, control beliefs may moderate the relationship between cardiovascular fitness and exercise adherence, as well as the relationship between subjective exercise experience and exercise adherence.

Previous research has found that subjective exercise experiences have a positive impact on exercise adherence. When individuals have positive experiences during exercise, such as feeling energized, accomplished and happy, they are more likely to continue exercising in the future, thus increasing their willingness to participate in sport [17]. Therefore, subjective exercise experience, as a self-evaluation of emotional feelings, has been suggested as a mediator between physical activity and exercise adherence [18]. Therefore, creating a positive and personalized exercise experience is an effective strategy to promote exercise adherence among college students. Thus, subjective exercise experiences may moderate the relationship between control beliefs and cardiovascular health. Exercise adherence refers to the extent to which an individual consistently engages in physical activity or exercise over an extended period of time [19]. It has a number of health benefits, including improved cardiovascular health, reduced risk of chronic diseases, better weight control and improved mental health [20]. Exercise adherence can be influenced by many factors such as motivation, exercise experience, social support and environmental factors. Therefore, designing an enjoyable, effective and achievable exercise programme, setting goals for exercise, monitoring, and providing feedback are important to promote exercise adherence.

**Fig. 1:**
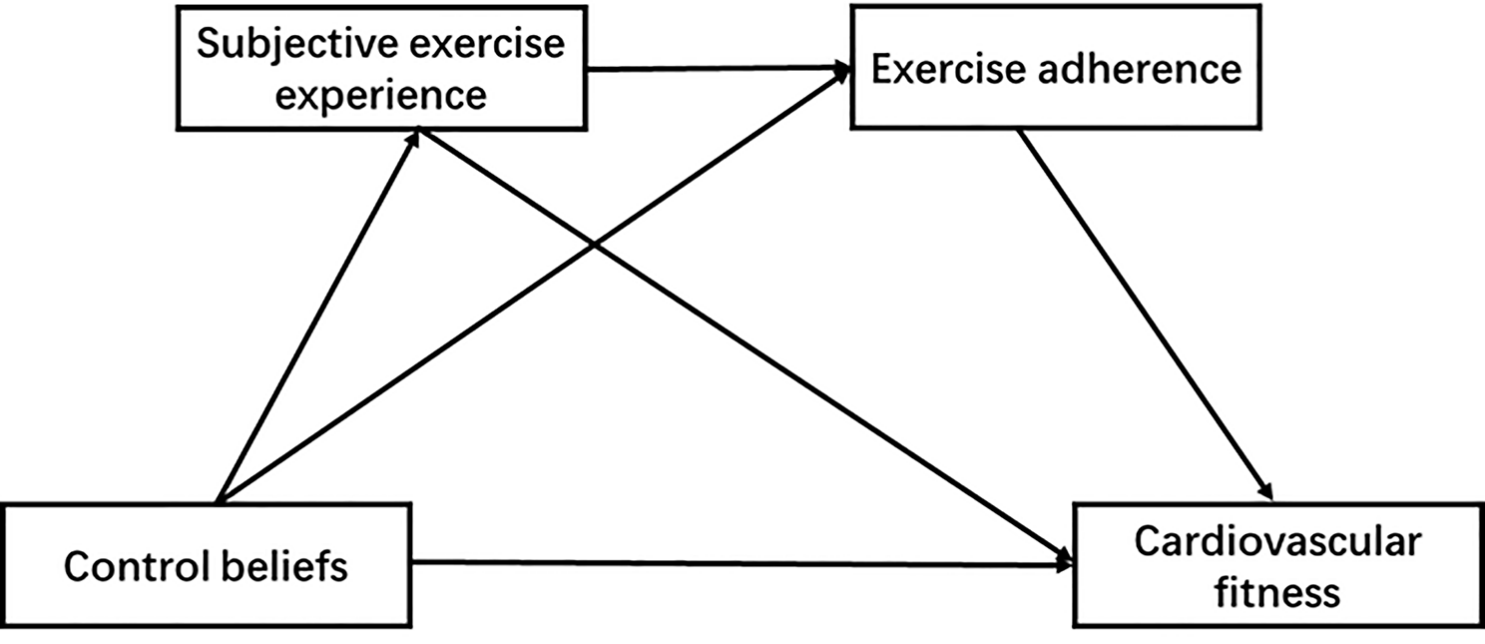
Moderated mediation model of the effect of control beliefs on cardiovascular fitness

Few previous studies have explored the effects and mechanisms of control beliefs on college students’ cardiovascular health based on the interlocking mediating effects of subjective exercise experience and exercise adherence. Introducing mediating variables such as subjective exercise experience and exercise adherence can reveal the effects of control beliefs on college students’ cardiovascular health. Taken together, the aims of this study were threefold. First, we tested whether cardiovascular fitness was significantly associated with control beliefs. Second, the present study explored whether subjective exercise experience and exercise adherence would act as mediators between cardiovascular fitness and control beliefs. Third, we tested whether subjective exercise experience and exercise adherence would act as chain mediators between cardiovascular fitness and control beliefs (Fig. 1). Based on the literature review, we proposed the following hypotheses:

### Hypothesis 1

The control beliefs of college students are positively related to cardiovascular fitness.

### Hypothesis 2

The subjective exercise experience among college students will act as a mediator between cardiovascular fitness and control beliefs.

### Hypothesis 3

The exercise adherence among college students will act as a mediator between cardiovascular fitness and control beliefs.

### Hypothesis 4

The subjective exercise experience and exercise adherence among college students will jointly act as a chain mediator role in the relationship between cardiovascular fitness and control beliefs.

## Participants and methods

### Participants

Using a cross-sectional survey research design, this study used a cluster random sampling method to conduct a sample survey. College students were instructed to take an online e-questionnaire group test with the assistance of the teachers of the sampled classes in the two colleges in China, and they completed a series of online e-questionnaires within 15 min and checked the submission status on site. A total of 2189 questionnaires were collected, and a total of 1854 valid questionnaires were obtained by eliminating invalid questionnaires in various ways, such as regular responses, reverse question quizzes, and filling time. Among them, 814 (43.9%) were males and 1040 (56.1%) were females. 1202 (64.8%) were rural households, and 652 (35.2%) were urban households. 1,181 college students (63.7%) were freshmen, and 670 college students (36.1%) were sophomores. As for the results of the last physical fitness test, 86 college students (4.6%) scored 60 or below, 490 college students (26.4%) scored 61–70, 561 college students (30.3%) scored 71–80, 557 college students (30.0%) scored 81–90, and 160 college students (8.6%) scored 91–100. College students and their parents provided written informed consent for this study.

### Measuring tools

#### Control beliefs scale (PARS-3)

The Control Beliefs Scale is a 4-item scale that was revised by Marsh et al [21]. The activity self-concept represents a person’s beliefs in control over regular physical activity, and “physical activity” in the subscale was revised to “exercise “, such as “I do physical activity (e.g., jogging, dancing, bicycling, aerobics, fitness, or swimming) at least three times a week”, which consisted of four questions and was scored on a 5-point Likert scale, ranging from “very unlikely” to “very likely”. The Likert 5 scale was used, ranging from 1 to 5 points. A confirmatory factor analysis (CFA) yielded acceptable fit indicators (NFI = 1.0, GFI = 1.0, CFI = 1.0, RMSEA = 0.04), and the Cronbach’s alpha for all questions in this study was 0.907. The reliability index and cultural adaptation of the scale applied in research on Chinese samples are well established [22]. The scores of each question on the scale were summed, and higher scores indicated higher control beliefs among college students.

#### Subjective exercise experience scale

The Subjective Exercise Experience Scale, revised by Paolin Dong [23], consists of 8 questions and is scored on a 5-point Likert scale, ranging from 1 to 5 on a scale of “very non-conforming” to “very conforming”. A confirmatory factor analysis (CFA) yielded acceptable fit indicators (NFI = 0.93, GFI = 0.96, CFI = 0.95, RMSEA = 0.05), and the Cronbach’s alpha of all the questions in this study was 0.928. The reliability index and cultural adaptation of the scale applied in research on Chinese samples are well established. The scores of each question on the scale were summed, and the higher the score, the higher the subjective exercise experience of college students.

#### Exercise adherence scale (PSSS)

The exercise adherence scale is a 6-item scale that was revised by Liu et al [24]. In this study, “outdoor sports” or “sports” on the scale were transformed into “exercise”. The scale consists of six questions and is scored on a 5-point Likert scale, ranging from 1 to 5 on a scale of strongly disagreeing to strongly agreeing. A confirmatory factor analysis (CFA) yielded acceptable fit indicators (NFI = 0.97, GFI = 0.99, CFI = 0.98, RMSEA = 0.04), and the Cronbach’s alpha of all the questions in this study was 0.941. The scores of each question on the scale were summed, and the higher the score, the higher the subjective exercise experience of college students. The survey was pilot-tested with 30 participants who were ultimately excluded from the survey, with some minor changes to the wording of the questionnaires and scales.

#### Cardiovascular fitness

Cardiovascular fitness is a measure of the cardiovascular system’s ability to efficiently supply oxygen to the body during exercise [25]. The cardiovascular fitness data used in this study were derived from the 800- and 1000-meter tests of the Physical Fitness Test for University Students. Physical fitness testing for university students refers to a systematic activity to assess the physical condition and measure the health level of university students. Physical fitness tests for university students usually include several indicators and test items, such as height, weight, lung capacity, heart rate, blood pressure, flexibility, strength, and so on. These indicators cover the assessment of body composition, cardiorespiratory fitness, muscular strength, and physical flexibility. Such tests are usually conducted on occasions such as physical education classes, physical assessment classes, and health promotion activities organized by schools or relevant organizations. In China, the cardiovascular fitness measurement was based on the indexes provided by the National Student Physical Health Standard (2014 Revised Edition). The National Student Physical Health Standard is an educational tool to promote the healthy development of students’ physical fitness and to motivate them to be physically active, which was used as the basis for the measurement of 1000 m for males and 800 m for females. The test results of male and female are divided into five grades, and the excellent score of male and female is ≤ 3′27″or 3′30″, 3′28″ to 3′42″ or 3′31″ to 3′44″, the average for male and female is 3′43″ to 4′07″ or 3′45″to 4′09″, male and female pass 4′08″ to 4′32″ or 4′10″ to 4′34″, failing ≥ 4′33″ or 4′35″, respectively. Score higher, indicating that cardiovascular fitness is better.

### Data analysis

SPSS 23.0 software was used to perform descriptive statistics and correlation analysis of the questionnaire data, and the model was tested for mediating effects using the PROCESS macros program, 4.0 Supplemented Process Models 4 and 15 macros for SPSS were used to test the mediation and moderated mediation models with 5000 random sample bootstrapping confidence intervals. All variables were standardized before being analyzed. Correlation analysis was used to explore the correlation between control beliefs, subjective exercise experience, exercise adherence, and cardiovascular fitness. Linear regression was used to explore the predictive effects of control beliefs on cardiovascular fitness and the mediating effects of subjective exercise experience and exercise adherence on control beliefs and cardiovascular fitness among college students.

## Results

### Correlation analysis

College students’ cardiovascular fitness were significantly and positively correlated with their control beliefs (*r* = 0.24), subjective exercise experience (*r* = 0.201), and exercise adherence(*r* = 0.269) (*p* < 0.01) (see Table 1). College students’ control beliefs and subjective exercise experience (r = 0.512), and exercise adherence (*r* = 0.774), subjective exercise experience and exercise adherence (*r* = 0.595) were significantly positively correlated with cardiovascular fitness. Therefore, Hypothesis 1 was supported. In addition, gender, grade, previous physical test were used as covariates in this study to exclude their influence on the results of the subsequent study.

**Table 1:** Statistics of Pearson Correlation Coefficient

	M ± SD	1	2	3	4	5	6	7
1.Gender	0.56 ± 0.49	1						
2.Grade	1.37 ± 0.49	0.088**	1					
3.Previous physical test	3.12 ± 1.04	0.090**	0.307**	1				
4.Control beliefs	12.87 ± 4.12	− 0.267**	-0.031	0.224**	1			
5.Subjective exercise experience	32.57 ± 6.31	− 0.179**	0.090**	0.253**	0.512**	1		
6.Exercise adherence	19.86 ± 5.7	− 0.212**	0.044	0.273**	0.774**	0.595**	1	
7.Cardiovascular fitness	64.4 ± 17.79	0.117**	0.067**	0.163**	0.240**	0.201**	0.269**	1

Notes: **p < 0.01

**Table 2:** Regression analysis of the multiple mediation model

Dependent variable	Independent variable	*R*	*R* ^*2*^	*F*	*β*	*t*	*P*
Cardiovascular fitness		0.321	0.103	42.301			
	Gender				0.170	7.752	0.000
	Grade				0.033	1.465	0.143
	Previous physical test				0.071	3.063	0.002
	Control beliefs				0.267	11.405	0.000
SEE		0.541	0.293	152.799			
	Gender				-0.068	-3.530	0.000
	Grade				0.064	3.259	0.001
	Previous physical test				0.128	6.248	0.000
	Control beliefs				0.452	21.986	0.000
Exercise adherence		0.811	0.657	590.652			
	Gender				-0.006	-0.408	0.683
	Grade				0.021	1.489	0.137
	Previous physical test				0.060	4.081	0.000
	Control beliefs				0.631	38.136	0.000
	SEE				0.260	15.593	0.000
Cardiovascular fitness		0.345	0.119	35.552			
	Gender				0.178	8.147	0.000
	Grade				0.023	1.032	0.302
	Previous physical test				0.049	2.082	0.038
	Control beliefs				0.121	3.458	0.001
	SEE				0.059	2.115	0.035
	Exercise adherence				0.160	4.363	0.000

Abbreviations: SEE, subjective exercise experience

### Regression analysis of control beliefs, SEE, exercise adherence, and cardiovascular fitness

The mediating effects of subjective exercise experience and exercise adherence between control beliefs and cardiovascular fitness among college students were examined, controlling for gender, grade, and previous physical test scores. The results showed (see Table 2) that the positive predictive effect of control beliefs on cardiovascular fitness was significant (β = 0.267, *P* < 0.001) and remained significant after the mediating variables (subjective exercise experience and exercise adherence) were included (β = 0.121, *P* < 0.01). Control beliefs had a significant positive predictive effect on subjective exercise experience (β = 0.452, *P* < 0.001) and exercise adherence (β = 0.631, *P* < 0.001), and subjective exercise experience had a significant positive predictive effect on exercise adherence among college students (β = 0.260, *P* < 0.001).

**Table 3:** Test results of Bootstrap mediation effect

		*Boot*	*BootCI*	*BootCI*	
Effect path	Effect value	Standard error	Lower limit	Upper limit	Effect percentage
Total effect	0.267	0.027	0.213	0.318	100.00%
Direct effect	0.121	0.034	0.054	0.188	45.32%
Total mediating effect	0.146	0.027	0.094	0.200	54.68%
CB-SEE-CF(Ind1)	0.026	0.015	-0.002	0.055	9.73%
CB-EA-CF(Ind2)	0.101	0.027	0.050	0.153	37.83%
CB-SEE-EA-CF(Ind3)	0.019	0.005	0.009	0.028	7.12%
C1(Ind1-Ind2)	-0.074	0.036	-0.144	-0.004	
C2(Ind1-Ind3)	0.008	0.017	-0.025	0.042	
C3(Ind2-Ind3)	0.082	0.022	0.041	0.127	

Abbreviations: CB, control beliefs; SEE, subjective exercise experience; EA, exercise adherence; CF, cardiovascular fitness

### Mediating effects test for SEE and EA between control beliefs and cardiovascular fitness

We used model 6 in the SPSS macro process to test hypothesis 3. The results are presented in Table 3. For the direct effects, the moderated mediation model showed that control beliefs were positively associated with cardiovascular fitness (β = 0.121, *p* < 0.01, 95% CI [0.054, 0.188]), indicating that control beliefs had a significant effect on college students’ cardiovascular fitness, with an effect value of 0.121 and a direct effect of 45.32%. In terms of the mediating effect, The path of " CB-SEE-CF " (β = 0.026, *p*>0.05, 95% CI [-0.002, 0.055]), which contains 0 in the upper and lower limits, the mediating effect of subjective exercise experience between control beliefs and cardiovascular fitness was not significant, the effect value was 0.026 and the mediating effect was 9.73%; The path of " CB-EA-CF " (β = 0.101, *p* < 0.01, 95% CI [0.050, 0.153]), the mediating effect of exercise adherence between control beliefs and cardiovascular fitness was significant, the effect value was 0.101and the mediating effect was 37.83%; The path of " CB-SEE-EA-CF " (β = 0.019, *p* < 0.01, 95% CI [0.009, 0.028]), the mediating effect of subjective exercise experience and exercise adherence between control beliefs and cardiovascular fitness was significant, the effect value was 0.019 and the mediating effect was 7.12%. In the comparison of mediating effects, the chain mediating effect value of subjective exercise experience and exercise adherence was lower than the independent mediating effect of both, and there was a statistical difference between it and the independent mediating effect of exercise adherence (bootstrap 95% confidence interval [0.041, 0.127]). In addition, the independent mediated effect value for subjective exercise experience was lower than the independent mediated effect for exercise adherence, and it was statistically different from the independent mediated effect for exercise adherence (bootstrap 95% confidence interval [-0.144, -0.004]).

## Discussion

Our research explored the relationship between college students’ control beliefs, subjective exercise experience, exercise adherence, and cardiorespiratory fitness through a cross-sectional design. The results showed that control beliefs were positively associated with cardiovascular fitness, and exercise adherence played a mediating role between control beliefs and cardiovascular fitness. Moreover, subjective exercise experience and exercise adherence played a moderating role between control beliefs and cardiovascular fitness.

### Influence of control beliefs on cardiovascular fitness

The results of this study validate H1, i.e., control beliefs can significantly predict the cardiovascular fitness of college students. In the field of sports, control beliefs are considered to be one of the important factors affecting individual exercise behavior. The results revealed that cardiovascular fitness can be increased by increasing control beliefs among college students. Our results verify several conclusions reached in previous studies. Research has shown that there is a relationship between control beliefs and cardiovascular fitness [13]. Several studies have examined the relationship between control beliefs and cardiovascular fitness in college students. For instance, college students who had higher control beliefs regarding their physical activity were more likely to engage in regular exercise and had better cardiovascular fitness levels [26]. A study found a significant positive correlation between control beliefs and enjoyment in physical activity, suggesting that when individuals feel in control of their physical abilities and outcomes, they are more likely to experience positive emotions [27]. Individuals with higher levels of cardiovascular fitness tend to have higher levels of control beliefs, which in turn can lead to better health outcomes. For example, individuals with higher control beliefs may be more likely to engage in regular exercise, maintain healthy eating habits, and seek out medical care when needed. In particular, Shen et al. studied the relationships between self-efficacy and cardiorespiratory fitness of 20,000 undergraduate students in China and found that cardiovascular endurance was associated with self-efficacy; college students with higher self-efficacy were more likely to have higher CF [16]. High self-efficacy might encourage female college students to actively participate in cardiovascular fitness [28]. When college students believe they can control their behavior and environment, such as by increasing exercise load, they are more likely to adopt active exercise strategies that improve exercise outcomes. Conversely, if college students lack control beliefs, they may adopt negative exercise strategies, such as reducing exercise load, thereby reducing exercise effectiveness. College students with strong control beliefs have stronger physical exercise adherence [29]. Therefore, control beliefs may have a positive effect on the cardiovascular fitness of college students.

### The mediating role of subjective exercise experience

The results of our study revealed that subjective exercise experience does not mediate between control beliefs and cardiovascular fitness. Our results do not test hypothesis 2. The subjective exercise experience of college students is also affected by unknown factors or variables such as exercise intensity, exercise duration, exercise mode, diet habit, biological nature, etc., which may lead to an insignificant mediating effect between control beliefs and cardiovascular fitness among college students. Specifically, when college students do proper exercise, they may experience pleasure, excitement, relaxation, or challenge. However, even with these positive subjective experiences, if college students lack or have insufficient control beliefs, they may still have problems such as laziness, lack of interest, and lack of willpower, resulting in lack of lasting exercise and ultimately affecting cardiovascular fitness. Similarly, even if college students have a strong belief in control, when their subjective exercise experience is not strong, exercise will no longer be sustained, ultimately affecting cardiovascular fitness. This situation may indicate that when exploring the relationship between college students’ control beliefs and cardiovascular fitness, subjective exercise experience needs to be studied together with other variables, so as to reveal whether such a mediating effect exists as well as the relevant mechanisms and influencing factors. In addition, corresponding research in a cross-sectional, whether it is a complete intermediary model or a partial intermediate decomposition model, stated that there is a deviation between the direct and intermediary effects. Therefore, follow-up studies should be conducted with an expanded sample size and an intervention study design.

### The mediating role of exercise adherence

The results of this study validate H3 as well; the mediation effect test showed that exercise adherence mediated the relationship between control beliefs and cardiovascular fitness. There is a positive correlation between exercise adherence and cardiovascular fitness. Wang suggests that college students should exercise regularly to increase their physical fitness [30]. Studies have shown that exercise adherence plays a mediating role between control beliefs and the cardiovascular fitness of college students. College students’ confidence in their ability to exercise can affect their exercise adherence and thus their cardiovascular fitness. Exercise adherence can lead to physiological adaptations [31] and improvements in cardiovascular fitness, such as increased cardiovascular endurance [32], improved oxygen delivery [33], and increased metabolic efficiency [34]. Specifically, there was a strong correlation between a college student’s belief in control and exercise adherence. When college students have a strong belief in self-confidence and control, they have a higher probability of continuing to exercise and show higher exercise adherence. Therefore, people with stronger control beliefs are more likely to stick to exercise in the long term and are more likely to maintain cardiovascular fitness [35]. In addition, exercise adherence may also affect cardiovascular fitness through other mechanisms. For example, long-term exercise can help the body gradually adapt to physical exercise and strengthen cardiopulmonary function and muscle strength, which can promote the cardiovascular fitness of the body [36]. In the United States, where encouraging regular physical activity is a major public health goal, results from the National Adolescent Health Survey show that adolescents who report 60 min of PA on 7 days have better levels of cardiovascular fitness than those who report ≤ 6 days and those who report 2 days [37]. In summary, exercise adherence is the ability to consistently engage in regular physical activity over time. To promote exercise adherence, it is important to consider personal factors, design an enjoyable and effective exercise program, and encourage regular feedback and modifications to the program.

### The chain effect of subjective exercise experience and exercise adherence between control beliefs and cardiovascular fitness

The fourth goal of this study is to explore the chain mediating role of subjective exercise experience and exercise adherence between control beliefs and cardiovascular fitness, and Hypothesis 4 has also been verified. Our study suggests that subjective exercise experience and exercise adherence play a mediating role between college students’ control beliefs and cardiovascular fitness. Subjective exercise experience is an evaluation of college students’ perceived exercise quality and satisfaction, which is represented by college students’ feelings and emotional experience exercise. According to environmental cognition theory, people’s perceptions and understandings of their environment internalize discriminative information about the behavioral environment, which stimulates emotional experiences and guides behavioral practices [38]. Feelings experienced during exercise have been identified as a primary factor in the decision to stop exercising [39]. Results from the SEES score show that adolescents who exercise regularly are significantly happier than those who don’t [40]. Subjective experience exercise is an important factor in determining exercise adherence. Research has shown that people who have a positive exercise experience are more likely to adhere to an exercise program in the long term. A negative exercise experience can lead to a lack of motivation and a reduced likelihood of continuing to exercise [41] .

College students’ subjective exercise experience can affect their exercise adherence and thus their cardiovascular fitness. When individuals adhere to a regular exercise routine, they are more likely to experience positive feelings and emotions associated with exercise, such as increased energy [42], improved mood [31], and reduced stress [43]. Additionally, consistent exercise can lead to improvements in physical fitness, which can further enhance the subjective exercise experience, highlighting the importance of establishing and maintaining a regular exercise routine for overall well-being [44]. Specifically, there is an interactive relationship between college students’ control beliefs, subjective exercise experience, and exercise adherence. When college students have a strong internal drive and are confident and motivated that they can achieve their goals, they can stimulate a higher level of motivation and interest in exercise, thereby improving the subjective experience exercise and promoting its persistence. Exercise adherence refers to an individual’s ability to exercise consistently, and continuous exercise can improve the body’s cardiovascular fitness [45]. Therefore, college students’ good subjective experience exercise can promote exercise adherence, resulting in a positive effect on cardiovascular fitness. In addition, through long-term exercise, the body can gradually adapt to exercise, and gradually enhance the adaptability of heart and lung function, muscle strength, and other aspects, so as to improve cardiovascular fitness. In conclusion, college students’ subjective exercise experience affects cardiovascular fitness through exercise adherence. When the subjective experience exercise is improved, this will increase his interest in exercise and their sense of self-efficacy, making him more likely to stick to the exercise, thus improving their cardiovascular fitness.

### Limitations

There are certain limitations to this study that should be mentioned. First and foremost, this study employed a cross-sectional design, which cannot give proof of causality. Second, as a source of research data for college students, this study relied solely on self-reported questionnaires, which may be prone to social desirability bias. Future research can use more objective measures of exposure and outcome variables. Third, this study only included college students, so further research is needed to see whether the findings extend to other populations, such as adults and adolescents.

Despite these limitations, the current work makes theoretical and practical advances. From a theoretical standpoint, this study builds on earlier research by stressing the significance of subjective exercise experience in influencing exercise adherence. Our work adds to the current understanding of the relationship between control beliefs and cardiovascular fitness in college students. From a practical standpoint, college departments should encourage college students to participate in physical activity while focusing on increasing subjective exercise experience and exercise adherence. Finally, it should take into account the individual disparities in control beliefs among students and provide unique social network functionalities for college users with various control beliefs.

### Suggestions

At the tertiary level, the concept of “health first” continues to be reinforced in the teaching of physical education, with more use of sports competitions to enhance the control beliefs of participation in cardiovascular fitness and to improve the subjective exercise experience, exercise adherence, and cardiovascular fitness of college students.

At the college student level, cardiovascular fitness should be enhanced by taking the initiative to establish stable cardiovascular fitness habits, incorporating cardiovascular fitness into daily studies, setting regular weekly cardiovascular fitness times and modalities such as running, cycling, or swimming, avoiding sedentary lifestyles, and gradually increasing the intensity and duration of exercise.

Although control beliefs have an impact on cardiovascular fitness, their influence is modest. As a result, further study is required, including bigger sample sizes and a greater focus on other factors influencing college students’ overall exercise adherence and cardiovascular fitness.

## Conclusions

In summary, control beliefs of college students can directly influence cardiovascular fitness status, as well as indirectly through the chain-mediated effects of subjective exercise experience and exercise adherence. This study is important in investigating how control beliefs are related to the cardiovascular fitness of Chinese college students, even if further replication and extension are needed. The motivational path can be described as follows. (1) Control beliefs can significantly and positively predict cardiovascular fitness; (2) control beliefs can indirectly influence cardiovascular fitness through the intermediary of exercise adherence; and (3) Subjective exercise experience and exercise adherence play a role in mediating the relationship between control beliefs and cardiovascular fitness. Based on the corresponding evidence, it was found that by improving college students’ control beliefs, they can strengthen their subjective exercise experience and exercise adherence, improve their cardiovascular fitness levels, and ultimately promote their physical fitness.

## Data Availability

The raw data supporting the conclusions of this article can be made available by the authors Rui Wang(wangrui_0529@163.com), without undue reservation.
